# Multiport quantum interferometry with universal nonunitary transformations

**DOI:** 10.1126/sciadv.aef4828

**Published:** 2026-09-30

**Authors:** Chaojie Wang, Yuning Zhang, Yuanyuan Chen, Xiubao Sui, Lixiang Chen

**Affiliations:** ^1^ Department of Physics, Xiamen University, Xiamen 361005, China.; ^2^ School of Electronic and Optical Engineering, Nanjing University of Science and Technology, Nanjing 210094, China.

## Abstract

Quantum interferometry provides a pivotal method for investigating complex photon-photon interaction, enabling various quantum information technologies that beyond the possibilities of classical physics. While such an effective interaction is mostly studied in *N* × *N* unitary transformation, *m* × *n* nonunitary transformation that relates to lossy multiport quantum interferometry requires a different approach. Here, we experimentally demonstrate a concise yet powerful tool to unravel fundamental features of multiport quantum interferometry with universal nonunitary transformations. To confirm their intriguing unitary and nonunitary properties, Hong-Ou-Mandel (HOM) interference is observed to reveal its nontrivial particle exchange phase, wherein the conventional HOM interference based on unitary transformation and the anomalous HOM interference based on nonunitary transformation can be readily harnessed. These results provide an alternative approach to investigate the quantum light-matter interaction, facilitating the use of complex multiport quantum interference for large-scale quantum information processing tasks such as quantum simulation and quantum computation.

## INTRODUCTION

The exploitation of quantum interference promises to enhance the performance of various quantum technologies that beyond the possibilities of classical physics, ranging from high-precision sensors (*1*, *2*) and intrinsically secure long-distance quantum communication security (*3*–*5*) to even providing exponential computational advantage for certain tasks (*6*, *7*). Hong-Ou-Mandel (HOM) interference is a prototypical example of such a quantum phenomenon that demonstrates the fact that identical photons arriving simultaneously at opposite input ports of a beam splitter would bunch into a common output as a direct result of their bosonic nature and the unitary property of lossless beam splitter (*8*–*10*). Because quantum technologies aim to provide speed up in several fields, complex and multiport quantum circuits are required (*11*, *12*). Among the now developed experimental platforms, unitary transformations are routinely modeled and used in optical quantum information processing, which conserves the number of photos and adhere to bosonic commutation relations. Backed by their implementation in terms of basic building blocks as phase shifters acting on individual modes and lossless beam splitter mixing two modes, any discrete unitary operator can be realized with a step-by-step implementation recipe. However, as unitarity imposes restrictions on the transformation matrix, nonunitarity may relax these restrictions and unlock fascinating opportunities for arbitrary transformations.

Because solving NP-complete problem using only unitary operators may be not feasible, nonunitary transformations have the potential to provide an alternative solution (*13*, *14*). In open physical systems with lossy time evolution, nonunitary operators prove useful in modeling their interaction with real environment and the inevitable imperfections of real optical elements (*15*). In contrast, as quantum entanglement is fragile and susceptible to decoherence, selective filtering of photonic quantum entanglement based on nonunitary optics is also developed to mitigate interaction between the quantum system and its deleterious environment (*16*). Another reason for stepping outside the framework of unitary networks is that transformations may have an unequal number of input and output mode of interest, which is a clear indicator of nonunitarity (*17*). Additionally, one noteworthy property of nonunitary transformation can allow for highly tunable quantum interference, and, thus, Boson sampling and quantum walks with such nonunitary transformation may potentially incorporate fermionic or anyonic behaviors that can be directly mimicked by reconfigurable multiport quantum interferometry (*12*, *18*).

Up to now, the experimental implementations of these unitary operations mainly focus on 2 × 2 matrix transformation. For example, lossy beam splitter based on graphene (*19*, *20*), metasurfaces (*21*), surface plasmon polaritons (*22*), Cr-SiN-Cr thin films (*23*), and fully integrated waveguides with engineered dissipation (*24*) have been extensively explored and shown to exhibit many intriguing phenomena. However, multiport interferometry with universal nonunitary transformation is essential for large-scale quantum information processing applications. In particular, programmable quantum chips have the stringent requirement for dynamical control of quantum operators. This problem has merely been theoretical investigated through the realization of arbitrary linear transformations, which provides an ingenious decomposition method to construct any desired operators (*17*, *25*). Thus, multiport quantum interferometry with universal nonunitary transformation has not yet been explored, particularly in terms of providing programmable functionality for integrated quantum photons.

Here, we show that any multiport interferometry with universal nonunitary transformation can be extracted from a photonic unitary circuit (*17*, *26*). Because any discrete unitary operator can be theoretically designed and experimentally realized through a decomposition strategy in terms of simple building blocks (*27*, *28*), the universal nonunitary operators are thus achievable in such a concise yet efficient manner. Moreover, the tunability of phase shifts and mode mixing provides precise control of individual elements of transformation matrix, resulting in the possibility of harnessing complex unitary and nonunitary operators that are necessary in programmable integrated optics. Building on the dilation from nonunitary to unitary operators and realization of any discrete unitary transformation, we use balanced beam splitters to construct a 4 × 4 unitary transformation, wherein the unitary property of contracted 2 × 2 transformation can be dynamically controlled. To confirm its intriguing particle exchange phase difference in unitary and nonunitary transformations, paired photons are incident on the 4 × 4 photonic circuit from different input modes such that the coalescence and anticoalescence of photons are determined by the contracted 2 × 2 operators. This highly tunable multiport quantum interference may provide an alternative approach to control photon-photon interaction, thereby providing a highly versatile mechanism for large-scale and complex quantum technologies.

This work validates a promising approach to use elaborate unitary circuits to extract any desired nonunitary transformation and set the stage for advanced quantum technologies to be developed on programmable photonic platforms. We envisage that incorporating such universal nonunitary transformation into the task of quantum information processing would substantially enrich the available quantum operations and quantum functionalities in photonic integrated circuits. For example, our method allows the construct of high-dimensional nonunitary transformation for multiphoton interference, which may provide an alternative route toward the experimental implementation of various quantum logic gates (*14*, *29*, *30*).

## RESULTS

Let us begin by outlining the basic method of implementing any nonunitary matrix $T_{m\times n}$ as shown in Fig. 1. Because complex Hadamard matrices relate the computational basis of a discrete Hilbert space to some mutually unbiased basis, $T_{m\times n}$ corresponds to *m* input modes and *n* output modes, whose physical implementation has relatively little explored. To tackle this issue, we extend the nonunitary operator $T_{m\times n}$ to unitary operator $S_{N\times N}$ through matrix dilation (*17*), wherein *N* represents the number of input and output modes, N≥n, and N≥m. Backed by an alternative arrangement of beam splitters and phase shifters, any unitary multiport interferometer can be achievable with optimal designs that have already been widely investigated (*27*, *28*). Thus, the formalism developed for unitary transformations describing lossless N×N interferometers is available to include any m×n nonunitary transformation. For example, we note that any m×n (m≤n) linear transformation can be either directly embedded within a 2n×2n unitary transformation within a scaling factor (*31*), or, in a more compact way, implemented by two separate m×n interferometers connected to each other via phase and amplitude modulators (*32*).

**Fig. 1.**
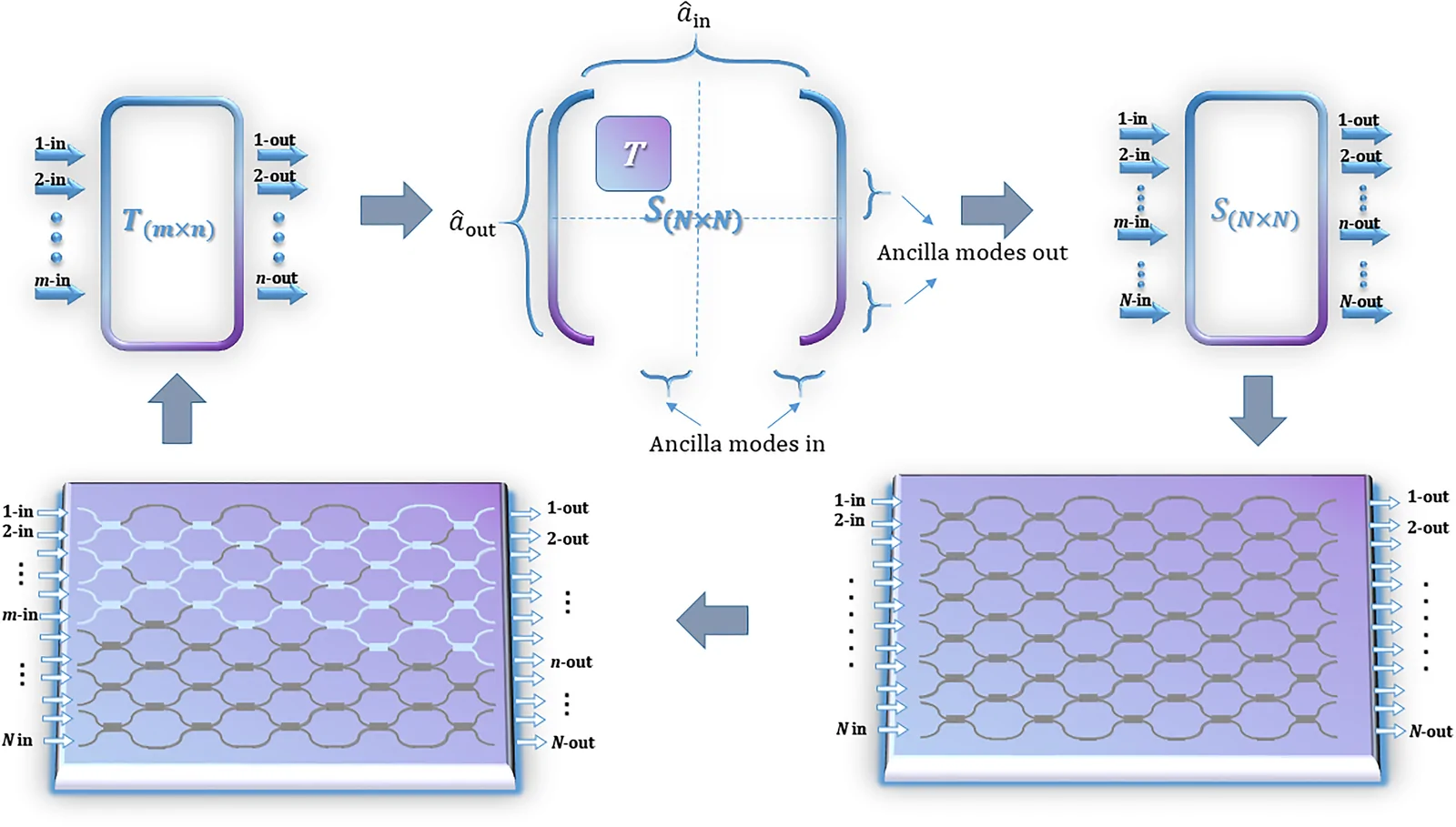
Schematic of realizing universal nonunitary transformation. The desired nonunitary matrix $T_{m\times n}$ can be embedded within a larger-scale unitary matrix $S_{N\times N}$ by following the matrix dilation (*17*). Because the implementation of $S_{N\times N}$ is available through a decomposition in terms of simple building blocks, $T_{m\times n}$ can be further extracted from this unitary photonic circuit.

For any target scattering matrix, a singular value decomposition is performed such that three main components—*U*, *D*, and *W*—are yielded. Through the analogous matrix dilation, the orthogonal matrices *U* and *W*, the diagonal matrix *D* can be implemented in a larger-scale multiport quantum interferometry (see the Supplementary Materials for a concrete example). In this work, we implement a proof-of-principle experiment that constructs the universal 2 × 2 nonunitary transformation. According to Naimark’s dilation theorem (*26*), this nonunitary matrix can be embedded within a 4 × 4 unitary matrix. As shown in Fig. 2, we use four balanced beam splitters and phase shifters controlled by relative time delays to realize a four-mode complex Hadamard unitary transformation as

$$S=\frac{1}{2}\left(\begin{array}{cccc} -e^{i\phi_{1}} & {\mathrm{ie}}^{i\phi_{1}} & {\mathrm{ie}}^{i\phi_{2}} & e^{i\phi_{2}}\\ {\mathrm{ie}}^{i\phi_{1}} & e^{i\phi_{1}} & -e^{i\phi_{2}} & {\mathrm{ie}}^{i\phi_{2}}\\ {\mathrm{ie}}^{i\phi_{3}} & -e^{i\phi_{3}} & e^{i\phi_{4}} & {\mathrm{ie}}^{i\phi_{4}}\\ e^{i\phi_{3}} & {\mathrm{ie}}^{i\phi_{3}} & {\mathrm{ie}}^{i\phi_{4}} & -e^{i\phi_{4}} \end{array}\right)\to \frac{1}{2}\left(\begin{array}{cccc} -1 & i & i & 1\\ i & 1 & -1 & i\\ i & -1 & 1 & i\\ 1 & i & i & -1 \end{array}\right)$$ (1)

where a single photon prepared in a well-defined position corresponding to an element of the computational basis, $\phi_{1/2/3/4}$ represents the shifted phase introduced by the translation stages in each spatial mode, respectively. In our experiment, we merely set all the phase shifts as 0 because the unstable phase drift limits the dynamical control of arbitrary phase shifts. Namely, this universal unitary scattering matrix becomes a simplified form. Thus, the selectively choosing of input and output modes may result in different unitary and nonunitary transformations. For example, if input modes 1 and 3 and output modes 1 and 4 are extracted, the resulting normalized 2 × 2 matrix becomes unitary $T_{13\&14}=\frac{1}{\sqrt{2}}\left(\begin{array}{cc} -1 & i\\ 1 & i \end{array}\right)$. In contrast, if input modes 1 and 3 and output modes 1 and 3 are extracted, the resulting normalized 2 × 2 matrix becomes nonunitary $T_{13\&13}=\frac{1}{\sqrt{2}}\left(\begin{array}{cc} -1 & i\\ i & 1 \end{array}\right)$. We envisage that the whole experimental setup can be implemented in photonic chip such that the relative phases between different spatial modes are inherently stable, and the integrated phase modulators would enable the on-demand construct of unitary and nonunitary transformation in a reconfigurable way (see the Supplementary Materials for more discussion).

**Fig. 2.**
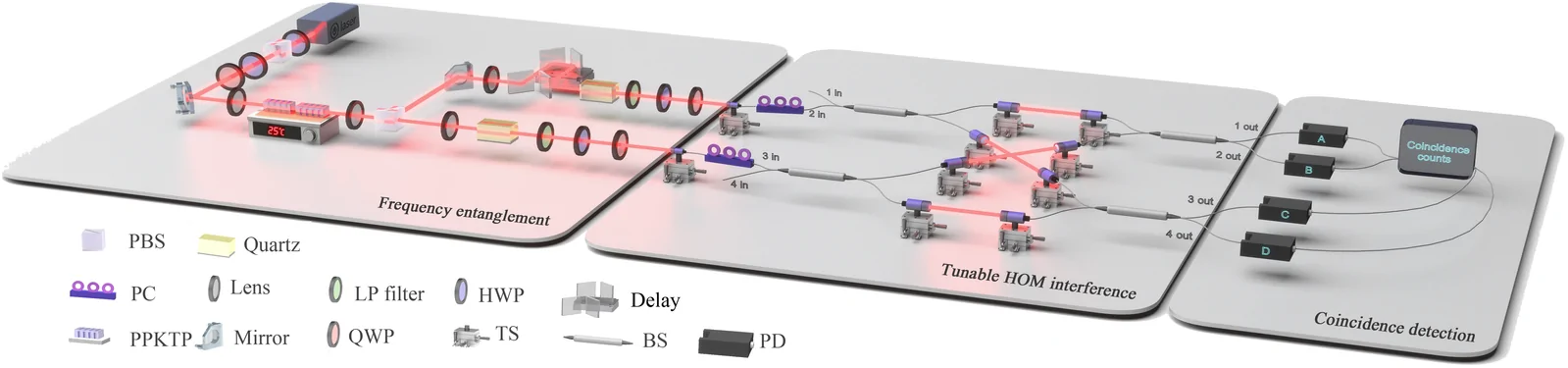
Experimental implementation of HOM interference with 2 × 2 unitary and nonunitary transformations, which are extracted from 4 × 4 unitary matrix by selectively choosing the input and output ports. PBS, polarizing beam splitter; Quartz, compensation crystal; PC, polarizing controller; LP filter, long-pass filter; PPKTP, periodically poled potassium titanyl phosphate crystal; QWP, quarter-wave plate; TS, translation stage; BS, in-fiber beam splitter; PD, single-photon detector.

One inherent difference between unitary and nonunitary operators is their particle exchange phases. To validate the tunability of the unitary property of 2 × 2 scattering matrix *T*, the contracted photonic circuit is used as the beam splitter in HOM interferometry such that quantum interference pattern would be determined by the particle exchange phase in scattering matrix. Without loss of generality, we express transformation matrix as $T\propto \left(\begin{array}{cc} t_{1} & r_{2}\\ r_{1} & t_{2} \end{array}\right)$, where the subscripts 1 and 2 represent different input modes, and their corresponding reflection and transmission coefficients are defined as $t_{1/2}=|t_{1/2}|\text{exp}\left(i\phi_{t_{1/2}}\right)$ and $r_{1/2}=|r_{1/2}|\text{exp}\left(i\phi_{r_{1/2}}\right)$, respectively. Thus, the output states can be derived from the transformation of the input states as

$$\begin{array}{c} {\hat{a}}_{\text{out}}=t_{1}{\hat{a}}_{\text{in}}+r_{2}{\hat{b}}_{\text{in}}+f_{a}{\hat{n}}_{a}\\ {\hat{b}}_{\text{out}}=r_{1}{\hat{b}}_{\text{in}}+t_{2}{\hat{a}}_{\text{in}}+f_{b}{\hat{n}}_{b} \end{array}$$ (2)

where $f_{a,b}$ denotes the lossy coefficient by following the noisy operator theory (*33*) and the noise operator ${\hat{n}}_{a,b}$ may exhibit loss or gain to preserve the commutators of the observable outputs for a generic beam splitter. Photon pairs entangled in the form of $|\Psi \rangle =\left(|\omega_{s}\omega_{i}\rangle +\text{exp}\left(\phi_{\omega }\right)|\omega_{i}\omega_{s}\rangle \right)/\sqrt{2}$ are incident on the beam splitter from opposite input modes. According to the transformations as shown in Eq. 2, the resultant state from different output ports becomes

$$\begin{array}{cc} {|\Psi \rangle }_{\text{out}}\propto & r_{1}r_{2}\left(|\omega_{s}^{a}\omega_{i}^{b}\rangle +\text{exp}\left(i\phi_{\omega }\right)|\omega_{i}^{a}\omega_{s}^{b}\rangle \right)\\ & +t_{1}t_{2}\left(\text{exp}\left(i\phi_{\omega }\right)|\omega_{s}^{a}\omega_{i}^{b}\rangle +|\omega_{i}^{a}\omega_{s}^{b}\rangle \right) \end{array}$$ (3)

where we disregard the events when one or two photons are out of this contracted 2 × 2 transmission matrix. Quantitatively, the normalized coincidence probabilities detected at the opposite output ports are expressed as

$$P\left(\tau \right)=\frac{1}{2}\left[1+\text{cos}\left(\Delta \tau +2\phi_{\text{pe}}+\phi_{\omega }\right)e^{-\sigma^{2}\tau^{2}}\right]$$ (4)

where τ indicates the relative time delay introduced in the imbalanced HOM interferometry, σ represents the RMS bandwidth of single photons, and $\phi_{\text{pe}}=\phi_{t_{1}}-\phi_{r_{1}}-\phi_{r_{2}}+\phi_{t_{2}}$ denotes the particle exchange phase that directly reveals the (non)unitary property of the 2 × 2 beam splitter.

Specifically, if the beam splitter exhibits as a unitary scattering matrix with $\phi_{\text{pe}}=\pi$, then identical photons and symmetrically entangled photons that arrive simultaneously on this unitary beam splitter from different input modes would bunch into a common output mode, which corresponds to the well-known HOM-dip as a direct result of bosonic nature. In contrast, if the beam splitter exhibits as a nonunitary scattering matrix with $\phi_{\text{pe}}=0$, then identical photons and symmetrically entangled photons that arrive simultaneously on this nonunitary beam splitter from different input modes would antibunch into opposite output modes, which corresponds to the anomalous HOM-peak that mimics the fermonic nature. Thus, HOM interference provides an observable certification for unitary and nonunitary property of the scattering matrix. Differently, for unitary beam splitter, antisymmetrically entangled photons with $\phi_{\omega }=\pi$ would behave as HOM-peak that results from their bosonic nature and unitary property of the scattering matrix. Analogously, for nonunitary beam splitter, antisymmetrically entangled photons with $\phi_{\omega }=\pi$ would behave as HOM-peak that also mimics the fermonic nature. Therefore, the selective input and output modes and tunable phase shifts can be used to implement programmable universal nonunitary transformations and provide a versatile tool to harness highly tunable quantum interference.

The experimental implementation is shown in Fig. 2, where two cascaded nonlinear crystals are used to create paired photon through the spontaneous parametric down conversion process, and their optical axes are mutually orthogonal for preparing tunable frequency entanglement. By adjusting its phase matching temperature, the frequency detuning $\Delta =|\omega_{s}-\omega_{i}|$ and relative phase $\phi_{\omega }$ can be continuously tuned, which provides a flexible method for creating the desired frequency entanglement. These paired photons are incident on the 4 × 4 quantum interferometry from different input modes. The photons that appear on four different output ports are identified by single-photon detectors labeled by A, B, C, and D. As a result, these coincidence events can be analyzed from different configurations of two random detectors (i.e., AB, AC, AD, BC, BD, and CD). By setting all the time delays at zero in this 4 × 4 multiport interferometry, the scattering matrix *S*_0_ is readily realized in our experiment. We note that more complex 4 × 4 matrix can also be achieved by phase stabilizations and shifts. Because we only create paired photons, different configurations of two random input modes and two random output modes are selected, which corresponds to a contracted 2 × 2 scattering matrix. For example, two input modes, 1-in and 3-in, and two output modes, 1-out and 3-out, would lead to a scattering matrix $T_{13\&14}=\frac{1}{\sqrt{2}}\left(\begin{array}{cc} -e^{i\phi_{1}} & {\mathrm{ie}}^{i\phi_{3}}\\ e^{i\phi_{2}} & e^{i\phi_{4}} \end{array}\right)$.

A precise scan of the relative time delay enables the direct observation of time-resolved two-photon interference patterns as shown in Figs. 3 to 5. By fitting the experimental results to theoretical prediction as shown in (*4*), we can obtain the corresponding frequency detunings in Figs. 4 and 5 as ∆=2.2 THz and ∆=4.2 THz, respectively. With respect to identical photons, their HOM interference patterns behave as dip or peak within the coherence time envelope, where the HOM-dip and HOM-peak are determined by unitary property of the extracted 2 × 2 scattering matrix. Because the selectively choosing of input and outputs relates to different unitary properties, their corresponding interference measurements may exhibit completely different patterns as shown in Fig. 3. Analogously, with respect to symmetrically and antisymmetrically entangled photons, their HOM interference patterns behave as periodic oscillation within the coherence time envelope, where the HOM-dip and HOM-peak at zero path delay are determined by both of the symmetric property of incident entangled photons and the unitary property of the extracted 2 × 2 scattering matrix. Therefore, the scattering matrix that arises from different input and output ports has tunable unitary property, and, thus, their interference measurements exhibit the corresponding patterns. Additionally, due to the symmetric property of incident entangled photons, the resultant HOM-dip and HOM-peak as shown in Fig. 4 are in analogy to those shown in Fig. 3, whereas the resultant HOM-dip and HOM-peak as shown in Fig. 5 are opposite to those shown in Fig. 3.

**Fig. 3.**
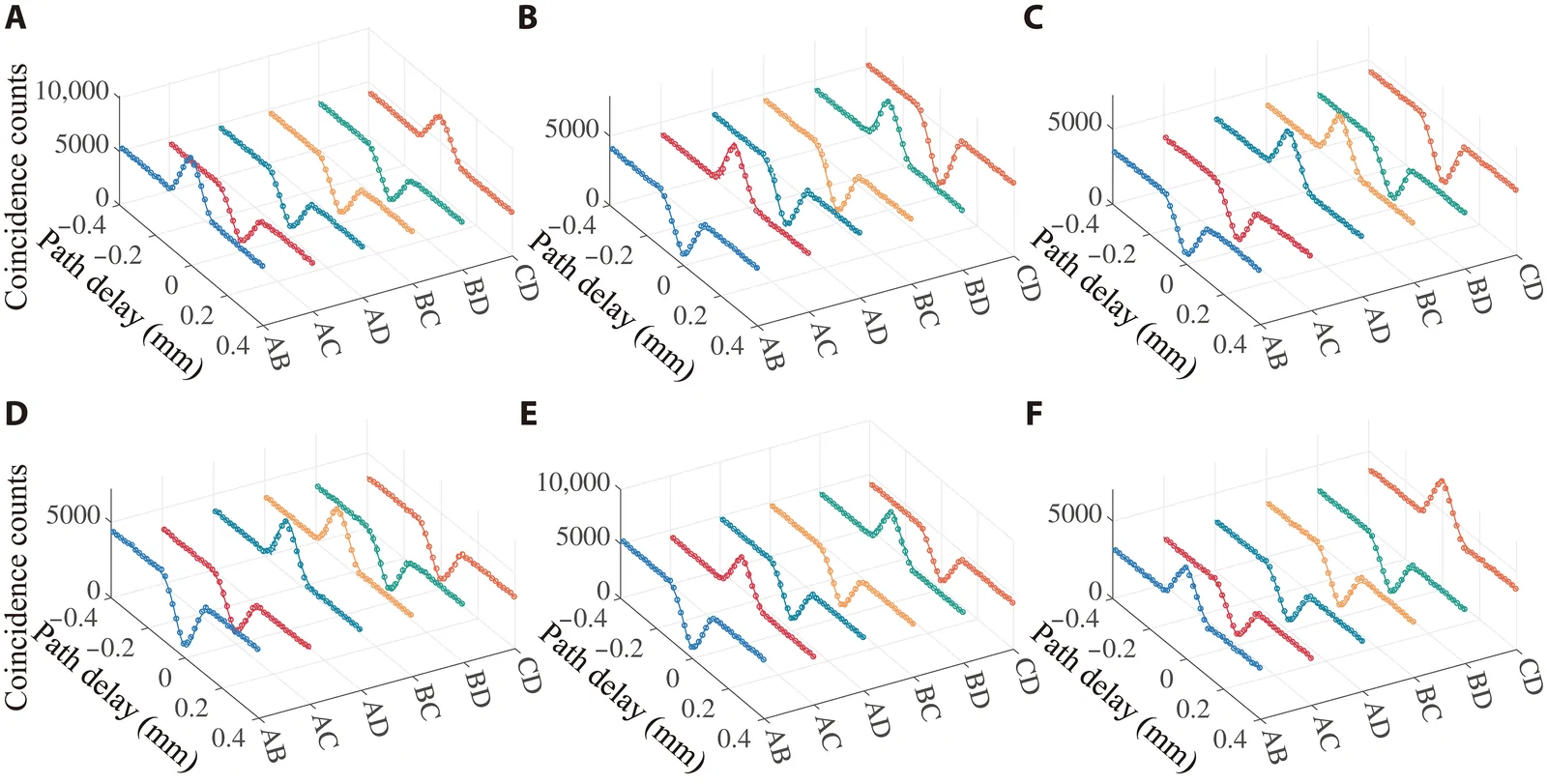
HOM interference of identical photons with 4 × 4 photonic interferometry. The input modes of incident photons are selected as (**A**) 1-in and 2-in, (**B**) 1-in and 3-in, (**C**) 1-in and 4-in, (**D**) 2-in and 3-in, (**E**) 2-in and 4-in, and (**F**) 3-in and 4-in.

**Fig. 4.**
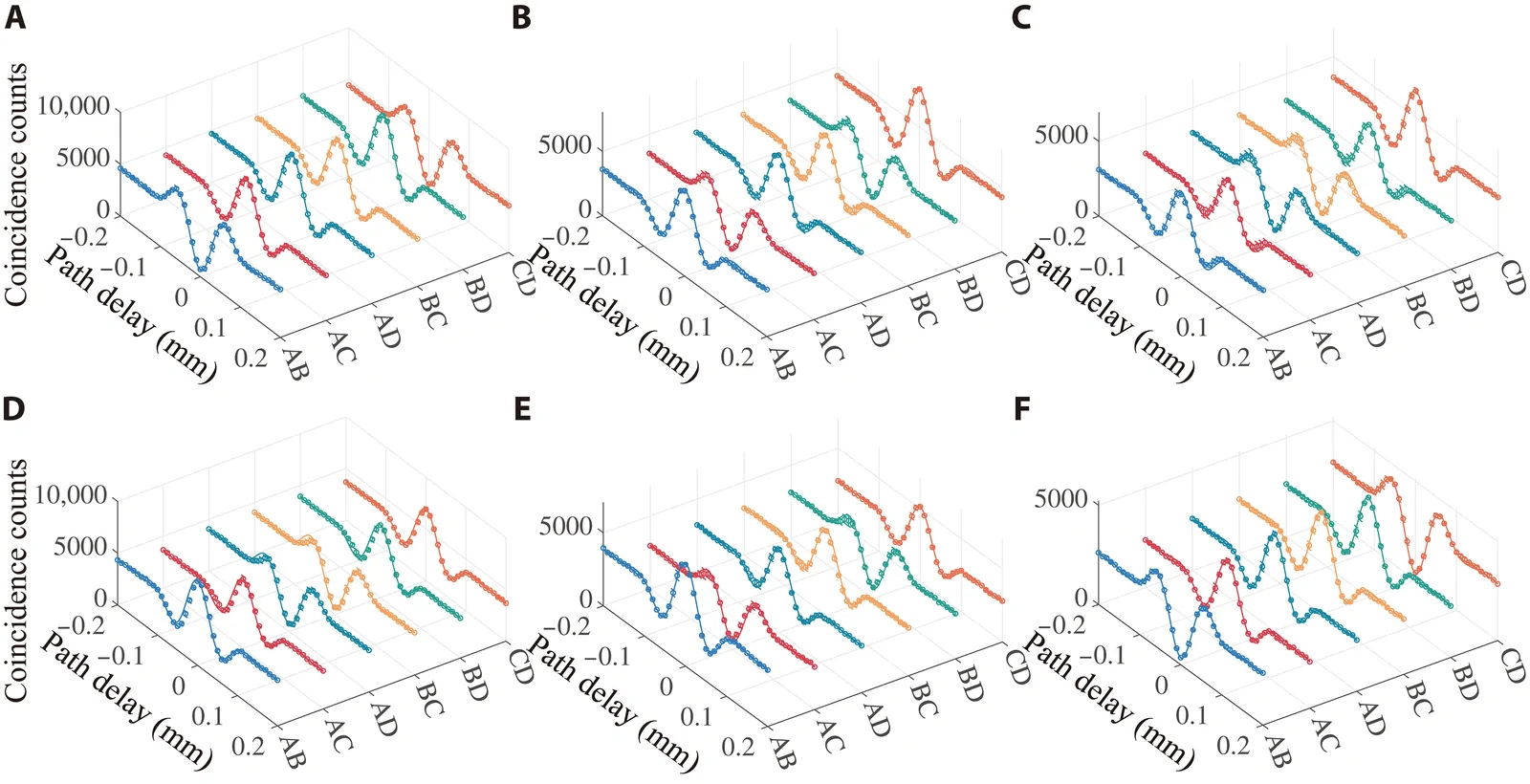
HOM interference of antisymmetrically entangled photons with 4 × 4 photonic interferometry, wherein the frequency detuning Δ = 2.2 THz. The input modes of incident photons are selected as (**A**) 1-in and 2-in, (**B**) 1-in and 3-in, (**C**) 1-in and 4-in, (**D**) 2-in and 3-in, (**E**) 2-in and 4-in, and (**F**) 3-in and 4-in.

**Fig. 5.**
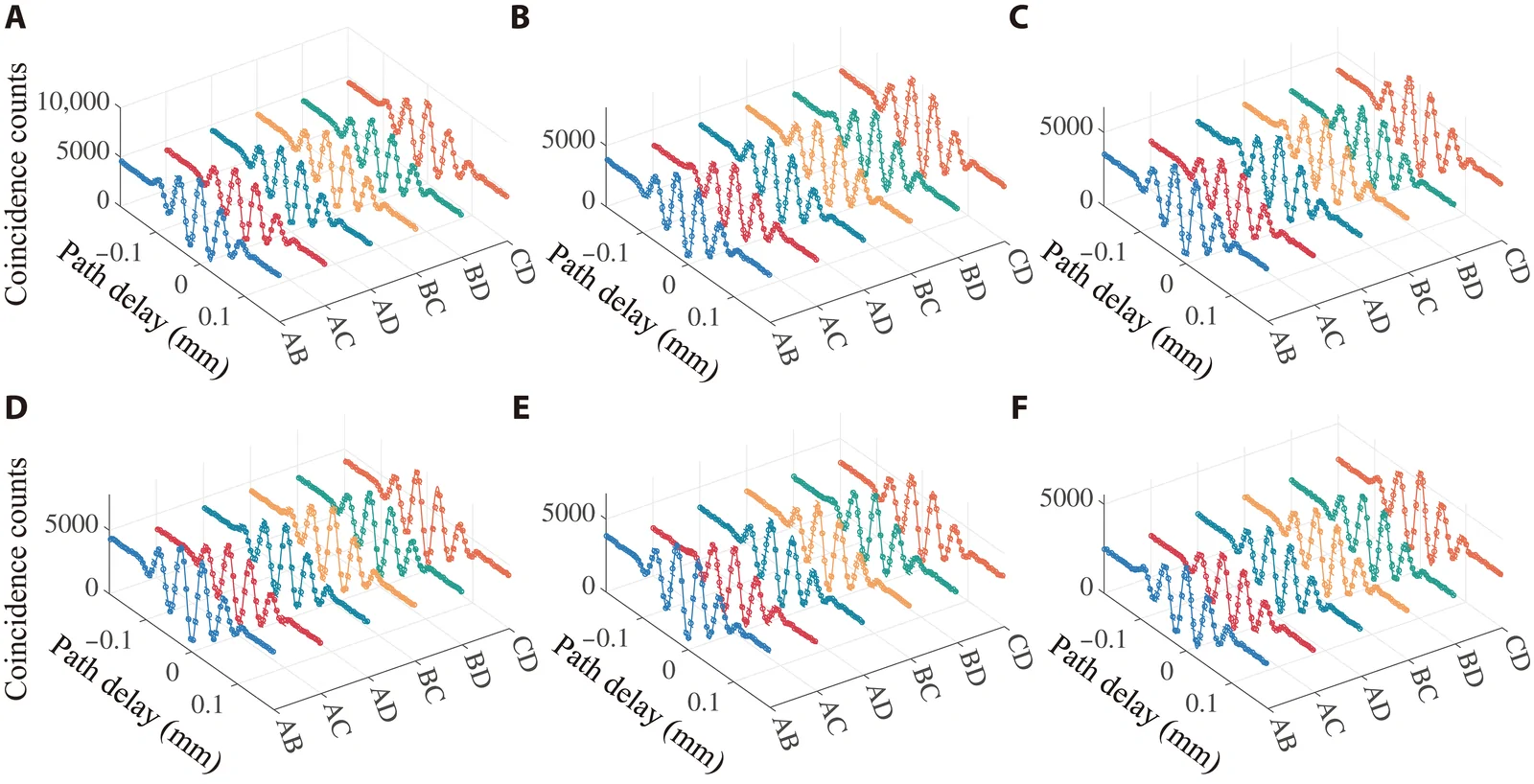
HOM interference of symmetrically entangled photons with 4 × 4 photonic interferometry, wherein the frequency detuning Δ = 4.2 THz. The input modes of incident photons are selected as (**A**) 1-in and 2-in, (**B**) 1-in and 3-in, (**C**) 1-in and 4-in, (**D**) 2-in and 3-in, (**E**) 2-in and 4-in, and (**F**) 3-in and 4-in.

Thanks to this concise yet efficient method based on conventional beam splitters and phase shifters, the specific visibilities of all measured HOM interference patterns are shown in Fig. 6. These experimental results reveal that the average visibility of HOM interference for frequency-degenerate, symmetric-entangled, and antisymmetric-entangled photons can reach 0.80 ± 0.02, 0.83 ± 0.04, and 0.81 ± 0.04, respectively. These visibilities may be further enhanced by optimizing the experimental implementation based on integration of multiport interferometer in photonic chips. While some existing scheme can implement loss-assisted nonunitary 2 × 2 scattering matrix, the coincidence events would be markedly decreased from lossy and nonunitary transformation, which is completely different from lossless and unitary transformation. This would pose a substantial limit for its applications in quantum simulation and quantum computation. Our scheme eludes this issue by using lossy unitary and nonunitary transformation. Therefore, the experimentally measured number of coincidence events almost keeps constant, where slight derivation can be attributed to the different efficiency of single-photon detectors and fiber couplings.

**Fig. 6.**
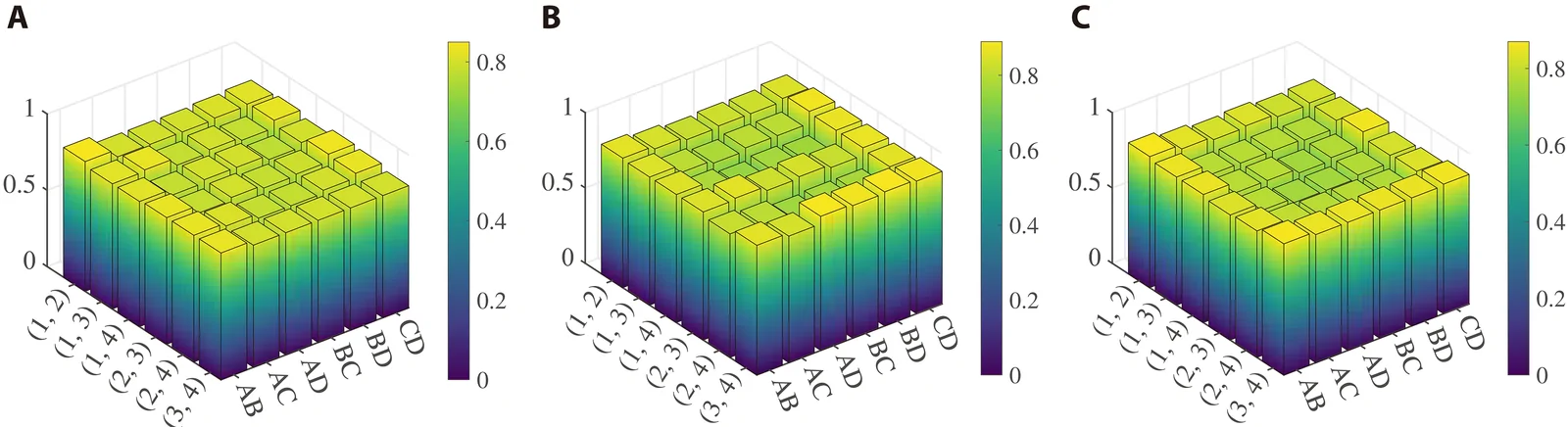
Visibility of HOM interference with 4 × 4 photonic interferometry. Experimental visibilities of HOM interference patterns as shown in (**A**) Fig. 3, (**B**) Fig. 4, and (**C**) Fig. 5, where (*i*, *j*) represent input ports *i*-in and *j*-in as shown in Fig. 2.

We note that our experimental four-input and four-output interferometry also allows for three- and four-photon interference, and the resultant quantum state can be continuously tuned by harnessing the photon loss, relative phase, input and output ports. For example, when four indistinguishable photons are simultaneously incident on four input ports of the experimental multiport quantum interferometry, the 4 × 4 scattering matrix as shown in (*7*) would transform the quantum state to $|\Psi \rangle =[e^{2i\left(\phi_{1}+\phi_{3}\right)}(\sqrt{6}|4000\rangle +\sqrt{6}|0400\rangle +2|2200\rangle )+$ $e^{2i\left(\phi_{2}+\phi_{4}\right)}(\sqrt{6}|0040\rangle +\sqrt{6}|0004\rangle +2|0022\rangle )-(e^{2i\left(\phi_{1}+\phi_{4}\right)}+e^{2i\left(\phi_{2}+\phi_{3}\right)})$ $(|2002\rangle -|2020\rangle -|0220\rangle +|0202\rangle +2|1111\rangle )-2i(e^{2i\left(\phi_{2}+\phi_{3}\right)}-$ $e^{2i(\phi_{1}+\phi_{4})})(|2011\rangle +|0211\rangle +|1102\rangle +|1120\rangle )]/8$, where *i*, *j*, *k*, and *l* are the symmetric superposition of the four-photon states corresponding to (*i*, *j*, *k*, *l*) photons exiting in the four output ports. When $\phi_{1}=\phi_{2}=\phi_{3}=\phi_{4}=0$, the four-photon quantum state is simplified as $|\Psi \rangle \to \sqrt{6}(|4000\rangle +|0400\rangle +|0040\rangle +|0004\rangle )/8+(|2200\rangle +|2020\rangle +|2002\rangle +|0220\rangle +|0202\rangle +|0022\rangle +|1111\rangle )/4$. However, when $\phi_{1}=\pi /2$, $\phi_{2}=\phi_{3}=\phi_{4}=0$, the four-photons quantum state is simplified as $|\Psi \rangle \to \sqrt{6}(|4000\rangle +|0400\rangle -|0040\rangle -|0004\rangle +$ $2|2200\rangle -2|0022\rangle )/8+\sqrt{2}i(|2011\rangle -|0211\rangle -|1120\rangle +|1102\rangle )/4$. These indicate the conventional multiphoton interference based on high-dimensional unitary transformation. To observe the anomalous multiphoton interference based on high-dimensional nonunitary transformation, a larger-scale multiport quantum interferometry is required. While our experimental realization reports the universal nonunitary transformation of paired photons, the overall scheme could be extended to mulitphoton interference such that the target high-dimensional unitary and nonunitary matrices can be constructed on demand.

## DISCUSSION

By embedding any nonunitary matrix within a larger-scale unitary matrix, we present a concise yet efficient method to deal with universal m×n nonunitary transformation. The selectively choosing of input and output ports allows for flexible control over the unitary property of transformation matrix, which is accompanied by the nontrivial particle exchange phase. To confirm this intriguing quantum phenomenon, we implement a HOM experiment to observe the particle exchange statistics. With respect to the conventional unitary operator, identical photons and symmetrically entangled photons exhibit the HOM-dip, and antisymmetrically entangled photons exhibit the HOM-peak, which corresponds to the well-known HOM interference patterns as a direct result of bosonic nature. In contrast, with respect to nonunitary operators, identical photons and symmetrically entangled photons exhibit the HOM-peak, and antisymmetrically entangled photons exhibit the HOM-dip, which corresponds to the anomalous HOM interference patterns that mimic the fermonic nature.

Notably, the tunable photon-photon interaction in multiport quantum interferometry has great potential in designing novel functions and complex quantum gate in integrated photonic circuits, particularly for those programmable quantum information tasks such as quantum simulation and quantum computation. Loss-induced nonunitary operator can naturally describe the interaction between open physical systems and may be connected to non-Hermitian systems that open avenues for quantum emulation and ultrasensitive metrology. Additionally, universal m×n nonunitary transformation can provide an opportunity for harnessing multiphoton and multimode quantum information processing, which allows for great benefit from the capability to introduce sophisticated nonunitary realization into integrated quantum optics and paves the way toward large-scale and programmable quantum information platforms.

## MATERIALS AND METHODS

In our experiment, we pump two mutually orthogonally oriented 2-mm-long type II periodically poled potassium titanyl phosphate crystals with a 405-nm continuous-wave grating-stabilized laser diode. The crystals provide type II collinear phase matching with the pump (p), signal (s), and idler (i) photons at center wavelengths of λ_p_ ≈ 405 nm and λ_s,i_ ≈ 810 nm at a temperature of 23.5°C. A pair of half-wave and quarter-wave plates in the pump beam are used to set the incident polarization at a diagonal state with a tunable phase shifter as $\left(|H_{p}\rangle +\text{exp}\left(i\phi_{\omega }\right)|V_{p}\rangle \right)/\sqrt{2}$, ensuring that both mutually orthogonal crystals are equally pumped. Consequently, the SPDC process in the first crystal yields the transformation of $|H_{p}\rangle \to |H_{\omega_{1}}V_{\omega_{2}}\rangle$ and, in the second crystal, produces the transformation of $|V_{p}\rangle \to |V_{\omega_{1}}H_{\omega_{2}}\rangle$, where *H* and *V* denote horizontal and vertical polarizations, respectively. Followed by a polarization beam splitter that routes orthogonal polarization states into distinct spatial modes, the resultant quantum state can be expressed as $|\Psi \rangle =\left(|\omega_{1}\omega_{2}\rangle +\text{exp}\left(i\phi_{\omega }\right)|\omega_{2}\omega_{1}\rangle \right)/\sqrt{2}$. The frequency detunings $\Delta =|\omega_{1}-\omega_{2}|$ and the relative phases $\phi_{\omega }$ can be continuously tuned by adjusting its phase matching temperature. By adjusting its phase matching temperature, the frequency detunings Δ and the relative phases $\phi_{\omega }$ can be continuously tuned from ∆=0 THz at 23.5°C (symmetric entanglement with degenerate wavelength) to ∆=2.2 THz and $\phi_{\omega }=\pi$ at 42°C (antisymmetric entanglement with nondegenerate wavelengths) and ∆=4.2 THz and $\phi_{\omega }=0$ at 60°C (symmetric entanglement with nondegenerate wavelengths).

Subsequently, these frequency-entangled or frequency-degenerate photons are coupled into single-mode fibers such that only the Gaussian mode can be transmitted into the 4 × 4 quantum interferometry from different input ports. We use four single-photon detectors labeled by A, B, C, and D to measure the probability of these photons appearing at four different output ports. The paired photons are detected by silicon avalanche photon diodes, and two-photon coincidence events are identified using a fast electronic AND gate when paired photons arrive at their corresponding detectors within a coincidence time window as 1 ns. As a result, these coincidence events can be analyzed from different configurations of two random detectors (i.e., AB, AC, AD, BC, BD, and CD).
