# Supplementary material for: Multiport quantum interferometry with universal nonunitary transformations
Source: Sci Adv. 2026 Sep 30;12(40):eaef4828. doi: 10.1126/sciadv.aef4828 (PMC13626082; doi:10.1126/sciadv.aef4828)
Supplement: Supplementary file 1 [file sciadv.aef4828_sm.pdf]

Science Advances
AAAS

Supplementary Materials for

Multiport quantum interferometry with universal nonunitary transformations

Chaojie Wang *et al.*

Corresponding author: Yuanyuan Chen, chenyy@xmu.edu.cn; Xiubao Sui, sxb@njust.edu.cn; Lixiang Chen, chenlx@xmu.edu.cn

Sci. Adv. **12**, eaef4828 (2026)
DOI: 10.1126/sciadv.aef4828

This PDF file includes:

Supplementary Text
Fig. S1
Tables S1 to S12

Supplementary Text

Modeling of Nonunitary BS and Design of photonic circuit

We use the singular value decomposition (SVD) method to construct nonunitary transformation matrices. This method allows us to decompose a general 2×2 linear transformation T into unitary matrices and a realvalued diagonal matrix as

$$T = UDW \tag{S1}$$

where U and W are unitary matrices, and D is a non-negative real diagonal matrix. For a nonunitary transformation matrix

$$T = \frac{1}{2}\begin{pmatrix} -1 & i \\ i & 1 \end{pmatrix} \tag{S2}$$

the results of SVD decomposition can be expressed in the following form

$$W = \frac{1}{\sqrt{2}}\begin{pmatrix} i & 1 \\ 1 & i \end{pmatrix}, D = \begin{pmatrix} 1 & 0 \\ 0 & 0 \end{pmatrix}, U = \frac{1}{\sqrt{2}}\begin{pmatrix} i & 1 \\ 1 & i \end{pmatrix}. \tag{S3}$$

Such a linear transformation can be achieved using auxiliary ports in the photonic circuits. To obtain the desired T in our scheme, we perform the following decomposition of a 4×4 matrix

$$S_T = S_U S_D S_W, \tag{S4}$$

where

$$S_U = \frac{1}{\sqrt{2}}\begin{pmatrix} i & 1 & 0 & 0 \\ 1 & i & 0 & 0 \\ 0 & 0 & i & 1 \\ 0 & 0 & 1 & i \end{pmatrix}, S_D = \begin{pmatrix} 1 & 0 & 0 & 0 \\ 0 & 0 & 1 & 0 \\ 0 & 1 & 0 & 0 \\ 0 & 0 & 0 & 1 \end{pmatrix}, S_W = \frac{1}{\sqrt{2}}\begin{pmatrix} i & 1 & 0 & 0 \\ 1 & i & 0 & 0 \\ 0 & 0 & i & 1 \\ 0 & 0 & 1 & i \end{pmatrix}. \tag{S5}$$

The S_T can be implemented using the photonic circuit as shown in Fig. S1. Taking all the phase shifts into account, the S_D can be re-written as

$$S_D = \begin{pmatrix} e^{i\phi_1} & 0 & 0 & 0 \\ 0 & 0 & e^{i\phi_2} & 0 \\ 0 & e^{i\phi_3} & 0 & 0 \\ 0 & 0 & 0 & e^{i\phi_4} \end{pmatrix}. \tag{S6}$$

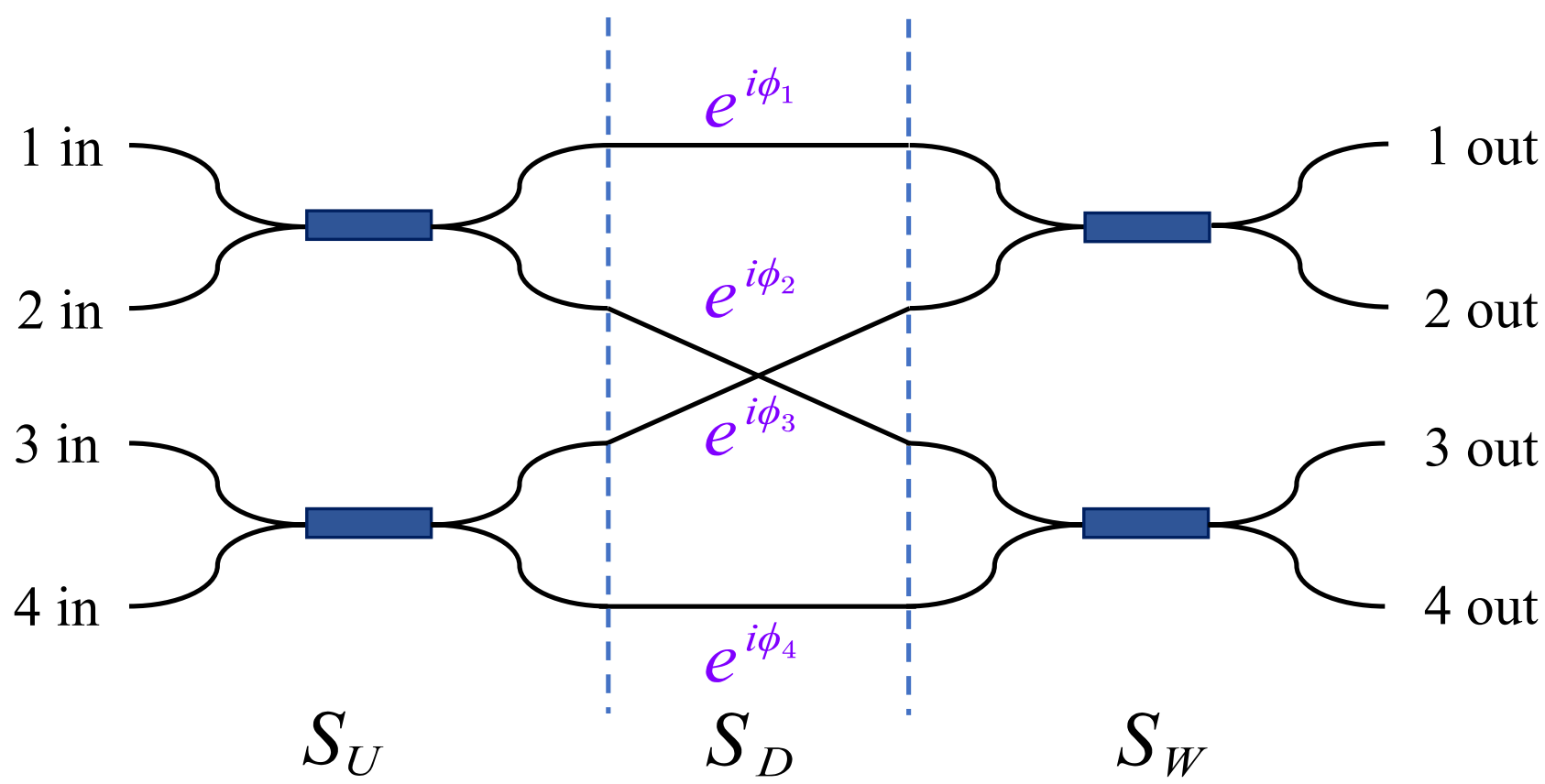

Figure S1: Conceptual diagram for implementation of a four-mode complex Hadamard unitary transformation. The 2×2 unitary and nonunitary transformations are extracted from the 4×4 unitary matrix including S_U, S_D and S_W by selectively choosing the input and output port.

Therefore, we can experimentally realize a four-mode complex Hadamard unitary transformation as

$$S_{4\times4} = \frac{1}{2}\begin{pmatrix} -e^{i\phi_1} & ie^{i\phi_1} & ie^{i\phi_2} & e^{i\phi_2} \\ ie^{i\phi_1} & e^{i\phi_1} & -e^{i\phi_2} & ie^{i\phi_2} \\ ie^{i\phi_3} & -e^{i\phi_3} & e^{i\phi_4} & ie^{i\phi_4} \\ e^{i\phi_3} & ie^{i\phi_3} & ie^{i\phi_4} & -e^{i\phi_4} \end{pmatrix}. \tag{S7}$$

HOM interference based on unitary and nonunitary transformations in 4×4 photonic interferometry

When $\phi_1 = \phi_2 = \phi_3 = \phi_4 = 0$, the universal nonunitary scattering matrix is simplified to

$$S_0 = \frac{1}{2}\begin{pmatrix} -1 & i & i & 1 \\ i & 1 & -1 & i \\ i & -1 & 1 & i \\ 1 & i & i & -1 \end{pmatrix}. \tag{S8}$$

Since we only prepare paired photons, the input and output modes are selected, which would result in unitary or nonunitary transformation matrix. Accordingly, their HOM interference patterns become HOM-dip or HOM-peak as a direct result of the unitary property of the transformation matrix.

Input modes	Output modes	Extracted scattering matrix	Unitary	HOM interference
1,2	1,2	$\frac{1}{\sqrt{2}}\begin{pmatrix} -1 & i \\ i & 1 \end{pmatrix}$	Nonunitary	HOM-peak
1,2	1,3	$\frac{1}{\sqrt{2}}\begin{pmatrix} -1 & i \\ i & -1 \end{pmatrix}$	Unitary	HOM-dip
1,2	1,4	$\frac{1}{\sqrt{2}}\begin{pmatrix} -1 & i \\ 1 & i \end{pmatrix}$	Unitary	HOM-dip
1,2	2,3	$\frac{1}{\sqrt{2}}\begin{pmatrix} i & 1 \\ i & -1 \end{pmatrix}$	Unitary	HOM-dip
1,2	2,4	$\frac{1}{\sqrt{2}}\begin{pmatrix} i & 1 \\ 1 & i \end{pmatrix}$	Unitary	HOM-dip
1,2	3,4	$\frac{1}{\sqrt{2}}\begin{pmatrix} i & -1 \\ 1 & i \end{pmatrix}$	Nonunitary	HOM-peak

Table S1: Theoretical prediction of HOM interference for input modes 1&2 based on the 4×4 transformation matrix S_0, whose experimental results are shown in Fig. 3(a).

Input modes	Output modes	Extracted scattering matrix	Unitary	HOM interference
1,3	1,2	$\frac{1}{\sqrt{2}}\begin{pmatrix} -1 & i \\ i & -1 \end{pmatrix}$	Unitary	HOM-dip
1,3	1,3	$\frac{1}{\sqrt{2}}\begin{pmatrix} -1 & i \\ i & 1 \end{pmatrix}$	Nonunitary	HOM-peak
1,3	1,4	$\frac{1}{\sqrt{2}}\begin{pmatrix} -1 & i \\ 1 & i \end{pmatrix}$	Unitary	HOM-dip
1,3	2,3	$\frac{1}{\sqrt{2}}\begin{pmatrix} i & -1 \\ i & 1 \end{pmatrix}$	Unitary	HOM-dip
1,3	2,4	$\frac{1}{\sqrt{2}}\begin{pmatrix} i & -1 \\ 1 & i \end{pmatrix}$	Nonunitary	HOM-peak
1,3	3,4	$\frac{1}{\sqrt{2}}\begin{pmatrix} i & 1 \\ 1 & i \end{pmatrix}$	Unitary	HOM-dip

Table S2: Theoretical prediction of HOM interference for input modes 1&3 based on the 4×4 transformation matrix S_0, whose experimental results are shown in Fig. 3(b).

Input modes	Output modes	Extracted scattering matrix	Unitary	HOM interference
1,4	1,2	$\frac{1}{\sqrt{2}}\begin{pmatrix} -1 & 1 \\ i & i \end{pmatrix}$	Unitary	HOM-dip
1,4	1,3	$\frac{1}{\sqrt{2}}\begin{pmatrix} -1 & 1 \\ i & i \end{pmatrix}$	Unitary	HOM-dip
1,4	1,4	$\frac{1}{\sqrt{2}}\begin{pmatrix} -1 & 1 \\ 1 & -1 \end{pmatrix}$	Nonunitary	HOM-peak
1,4	2,3	$\frac{1}{\sqrt{2}}\begin{pmatrix} i & i \\ i & i \end{pmatrix}$	Nonunitary	HOM-peak
1,4	2,4	$\frac{1}{\sqrt{2}}\begin{pmatrix} i & i \\ 1 & -1 \end{pmatrix}$	Unitary	HOM-dip
1,4	3,4	$\frac{1}{\sqrt{2}}\begin{pmatrix} i & i \\ 1 & -1 \end{pmatrix}$	Unitary	HOM-dip

Table S3: Theoretical prediction of HOM interference for input modes 1&4 based on the 4×4 transformation matrix S_0, whose experimental results are shown in Fig. 3(c).

Input modes	Output modes	Extracted scattering matrix	Unitary	HOM interference
2,3	1,2	$\frac{1}{\sqrt{2}}\begin{pmatrix} i & i \\ 1 & -1 \end{pmatrix}$	Unitary	HOM-dip
2,3	1,3	$\frac{1}{\sqrt{2}}\begin{pmatrix} i & i \\ -1 & 1 \end{pmatrix}$	Unitary	HOM-dip
2,3	1,4	$\frac{1}{\sqrt{2}}\begin{pmatrix} i & i \\ i & i \end{pmatrix}$	Nonunitary	HOM-peak
2,3	2,3	$\frac{1}{\sqrt{2}}\begin{pmatrix} 1 & -1 \\ -1 & 1 \end{pmatrix}$	Nonunitary	HOM-peak
2,3	2,4	$\frac{1}{\sqrt{2}}\begin{pmatrix} 1 & -1 \\ i & i \end{pmatrix}$	Unitary	HOM-dip
2,3	3,4	$\frac{1}{\sqrt{2}}\begin{pmatrix} -1 & 1 \\ i & i \end{pmatrix}$	Unitary	HOM-dip

Table S4: Theoretical prediction of HOM interference for input modes 2&3 based on the 4×4 transformation matrix S_0, whose experimental results are shown in Fig. 3(d).

Input modes	Output modes	Extracted scattering matrix	Unitary	HOM interference
2,4	1,2	$\frac{1}{\sqrt{2}}\begin{pmatrix} i & 1 \\ 1 & i \end{pmatrix}$	Unitary	HOM-dip
2,4	1,3	$\frac{1}{\sqrt{2}}\begin{pmatrix} i & 1 \\ -1 & i \end{pmatrix}$	Nonunitary	HOM-peak
2,4	1,4	$\frac{1}{\sqrt{2}}\begin{pmatrix} i & 1 \\ i & -1 \end{pmatrix}$	Unitary	HOM-dip
2,4	2,3	$\frac{1}{\sqrt{2}}\begin{pmatrix} 1 & i \\ -1 & i \end{pmatrix}$	Unitary	HOM-dip
2,4	2,4	$\frac{1}{\sqrt{2}}\begin{pmatrix} 1 & i \\ i & -1 \end{pmatrix}$	Nonunitary	HOM-peak
2,4	3,4	$\frac{1}{\sqrt{2}}\begin{pmatrix} -1 & i \\ i & -1 \end{pmatrix}$	Unitary	HOM-dip

Table S5: Theoretical prediction of HOM interference for input modes 2&4 based on the 4×4 transformation matrix S_0, whose experimental results are shown in Fig. 3(e).

Input modes	Output modes	Extracted scattering matrix	Unitary	HOM interference
3,4	1,2	$\frac{1}{\sqrt{2}}\begin{pmatrix} i & 1 \\ -1 & i \end{pmatrix}$	Nonunitary	HOM-peak
3,4	1,3	$\frac{1}{\sqrt{2}}\begin{pmatrix} i & 1 \\ 1 & i \end{pmatrix}$	Unitary	HOM-dip
3,4	1,4	$\frac{1}{\sqrt{2}}\begin{pmatrix} i & 1 \\ i & -1 \end{pmatrix}$	Unitary	HOM-dip
3,4	2,3	$\frac{1}{\sqrt{2}}\begin{pmatrix} -1 & i \\ 1 & i \end{pmatrix}$	Unitary	HOM-dip
3,4	2,4	$\frac{1}{\sqrt{2}}\begin{pmatrix} -1 & i \\ i & -1 \end{pmatrix}$	Unitary	HOM-dip
3,4	3,4	$\frac{1}{\sqrt{2}}\begin{pmatrix} 1 & i \\ i & -1 \end{pmatrix}$	Nonunitary	HOM-peak

Table S6: Theoretical prediction of HOM interference for input modes 3&4 based on the 4×4 transformation matrix S_0, whose experimental results are shown in Fig. 3(f).

When $\phi_1 = \pi$, and $\phi_2 = \phi_3 = \phi_4 = 0$, the universal nonunitary scattering matrix is simplified to

$$S_1 = \frac{1}{2}\begin{pmatrix} 1 & -i & i & 1 \\ -i & -1 & -1 & i \\ i & -1 & 1 & i \\ 1 & i & i & -1 \end{pmatrix}. \tag{S9}$$

Since these measurements has a strict requirement for phase stabilization, we cannot directly observe their HOM interference patterns in our experiment. However, we note that their corresponding experimental results can be readily observed in on-chip integrated photonic circuits. We envisage that this concise yet efficient method can provide an alternative tool for harnessing photon-photon interaction in larger-scale and programmable photonic platforms. Here, we list the theoretical prediction of HOM interference in the S_1 transformation matrix.

Input modes	Output modes	Extracted scattering matrix	Unitary	HOM interference
1,2	1,2	$\frac{1}{\sqrt{2}}\begin{pmatrix} 1 & -i \\ -i & -1 \end{pmatrix}$	Nonunitary	HOM-peak
1,2	1,3	$\frac{1}{\sqrt{2}}\begin{pmatrix} 1 & -i \\ i & -1 \end{pmatrix}$	Unitary	HOM-dip
1,2	1,4	$\frac{1}{\sqrt{2}}\begin{pmatrix} 1 & -i \\ 1 & i \end{pmatrix}$	Unitary	HOM-dip
1,2	2,3	$\frac{1}{\sqrt{2}}\begin{pmatrix} -i & -1 \\ i & -1 \end{pmatrix}$	Unitary	HOM-dip
1,2	2,4	$\frac{1}{\sqrt{2}}\begin{pmatrix} -i & -1 \\ 1 & i \end{pmatrix}$	Unitary	HOM-dip
1,2	3,4	$\frac{1}{\sqrt{2}}\begin{pmatrix} i & -1 \\ 1 & i \end{pmatrix}$	Nonunitary	HOM-peak

Table S7: Theoretical prediction of HOM interference for input modes 1&2 based on the 4×4 transformation matrix S_1.

Input modes	Output modes	Extracted scattering matrix	Unitary	HOM interference
1,3	1,2	$\frac{1}{\sqrt{2}}\begin{pmatrix} 1 & i \\ -i & -1 \end{pmatrix}$	Unitary	HOM-dip
1,3	1,3	$\frac{1}{\sqrt{2}}\begin{pmatrix} 1 & i \\ i & 1 \end{pmatrix}$	Unitary	HOM-dip
1,3	1,4	$\frac{1}{\sqrt{2}}\begin{pmatrix} 1 & i \\ 1 & i \end{pmatrix}$	Nonunitary	HOM-peak
1,3	2,3	$\frac{1}{\sqrt{2}}\begin{pmatrix} -i & -1 \\ i & 1 \end{pmatrix}$	Nonunitary	HOM-peak
1,3	2,4	$\frac{1}{\sqrt{2}}\begin{pmatrix} -i & -1 \\ 1 & i \end{pmatrix}$	Unitary	HOM-dip
1,3	3,4	$\frac{1}{\sqrt{2}}\begin{pmatrix} i & 1 \\ 1 & i \end{pmatrix}$	Unitary	HOM-dip

Table S8: Theoretical prediction of HOM interference for input modes 1&3 based on the 4×4 transformation matrix S_1.

Input modes	Output modes	Extracted scattering matrix	Unitary	HOM interference
1,4	1,2	$\frac{1}{\sqrt{2}}\begin{pmatrix} 1 & 1 \\ -i & i \end{pmatrix}$	Unitary	HOM-dip
1,4	1,3	$\frac{1}{\sqrt{2}}\begin{pmatrix} 1 & 1 \\ i & i \end{pmatrix}$	Nonunitary	HOM-peak
1,4	1,4	$\frac{1}{\sqrt{2}}\begin{pmatrix} 1 & 1 \\ 1 & -1 \end{pmatrix}$	Unitary	HOM-dip
1,4	2,3	$\frac{1}{\sqrt{2}}\begin{pmatrix} -i & i \\ i & i \end{pmatrix}$	Unitary	HOM-dip
1,4	2,4	$\frac{1}{\sqrt{2}}\begin{pmatrix} -i & i \\ 1 & -1 \end{pmatrix}$	Nonunitary	HOM-peak
1,4	3,4	$\frac{1}{\sqrt{2}}\begin{pmatrix} i & i \\ 1 & -1 \end{pmatrix}$	Unitary	HOM-dip

Table S9: Theoretical prediction of HOM interference for input modes 1&4 based on the 4×4 transformation matrix S_1.

Input modes	Output modes	Extracted scattering matrix	Unitary	HOM interference
2,3	1,2	$\frac{1}{\sqrt{2}}\begin{pmatrix} -i & i \\ -1 & -1 \end{pmatrix}$	Unitary	HOM-dip
2,3	1,3	$\frac{1}{\sqrt{2}}\begin{pmatrix} -i & i \\ -1 & 1 \end{pmatrix}$	Nonunitary	HOM-peak
2,3	1,4	$\frac{1}{\sqrt{2}}\begin{pmatrix} -i & i \\ i & i \end{pmatrix}$	Unitary	HOM-dip
2,3	2,3	$\frac{1}{\sqrt{2}}\begin{pmatrix} -1 & -1 \\ -1 & 1 \end{pmatrix}$	Unitary	HOM-dip
2,3	2,4	$\frac{1}{\sqrt{2}}\begin{pmatrix} -1 & -1 \\ i & i \end{pmatrix}$	Nonunitary	HOM-peak
2,3	3,4	$\frac{1}{\sqrt{2}}\begin{pmatrix} -1 & 1 \\ i & i \end{pmatrix}$	Unitary	HOM-dip

Table S10: Theoretical prediction of HOM interference for input modes 2&3 based on the 4×4 transformation matrix S_1.

Input modes	Output modes	Extracted scattering matrix	Unitary	HOM interference
2,4	1,2	$\frac{1}{\sqrt{2}}\begin{pmatrix} -i & 1 \\ -1 & i \end{pmatrix}$	Unitary	HOM-dip
2,4	1,3	$\frac{1}{\sqrt{2}}\begin{pmatrix} -i & 1 \\ -1 & i \end{pmatrix}$	Unitary	HOM-dip
2,4	1,4	$\frac{1}{\sqrt{2}}\begin{pmatrix} -i & 1 \\ i & -1 \end{pmatrix}$	Nonunitary	HOM-peak
2,4	2,3	$\frac{1}{\sqrt{2}}\begin{pmatrix} -1 & i \\ -1 & i \end{pmatrix}$	Nonunitary	HOM-peak
2,4	2,4	$\frac{1}{\sqrt{2}}\begin{pmatrix} -1 & i \\ i & -1 \end{pmatrix}$	Unitary	HOM-dip
2,4	3,4	$\frac{1}{\sqrt{2}}\begin{pmatrix} -1 & i \\ i & -1 \end{pmatrix}$	Unitary	HOM-dip

Table S11: Theoretical prediction of HOM interference for input modes 2&4 based on the 4×4 transformation matrix S_1.

Input modes	Output modes	Extracted scattering matrix	Unitary	HOM interference
3,4	1,2	$\frac{1}{\sqrt{2}}\begin{pmatrix} i & 1 \\ -1 & i \end{pmatrix}$	Nonunitary	HOM-peak
3,4	1,3	$\frac{1}{\sqrt{2}}\begin{pmatrix} i & 1 \\ 1 & i \end{pmatrix}$	Unitary	HOM-dip
3,4	1,4	$\frac{1}{\sqrt{2}}\begin{pmatrix} i & 1 \\ i & -1 \end{pmatrix}$	Unitary	HOM-dip
3,4	2,3	$\frac{1}{\sqrt{2}}\begin{pmatrix} -1 & i \\ 1 & i \end{pmatrix}$	Unitary	HOM-dip
3,4	2,4	$\frac{1}{\sqrt{2}}\begin{pmatrix} -1 & i \\ i & -1 \end{pmatrix}$	Unitary	HOM-dip
3,4	3,4	$\frac{1}{\sqrt{2}}\begin{pmatrix} 1 & i \\ i & -1 \end{pmatrix}$	Nonunitary	HOM-peak

Table S12: Theoretical prediction of HOM interference for input modes 3&4 based on the 4×4 transformation matrix S_1.

Four-photon interference in a 4×4 interferometer

When four indistinguishable photons are simultaneously incident on four input ports of the 4×4 quantum interferometry, the quantum state can be written as:

$$|\psi\rangle_{in} = \hat{a}^{\dagger}_{1\text{in}}\hat{a}^{\dagger}_{2\text{in}}\hat{a}^{\dagger}_{3\text{in}}\hat{a}^{\dagger}_{4\text{in}}\,|0000\rangle \tag{S10}$$

where $\hat{a}^{\dagger}_{i\text{in}}$ represents the creation operator for the i-th input port. After passing through the 4×4 interferometer, the output states can be written as:

$$\begin{aligned}|\psi\rangle_{out} = \frac{1}{16}&[-e^{i\phi_1}\hat{a}^{\dagger}_{1\text{out}} + ie^{i\phi_1}\hat{a}^{\dagger}_{2\text{out}} + ie^{i\phi_2}\hat{a}^{\dagger}_{3\text{out}} + e^{i\phi_2}\hat{a}^{\dagger}_{4\text{out}}]\\ &[ie^{i\phi_1}\hat{a}^{\dagger}_{1\text{out}} + e^{i\phi_1}\hat{a}^{\dagger}_{2\text{out}} - e^{i\phi_2}\hat{a}^{\dagger}_{3\text{out}} + ie^{i\phi_2}\hat{a}^{\dagger}_{4\text{out}}]\\ &[ie^{i\phi_3}\hat{a}^{\dagger}_{1\text{out}} - e^{i\phi_3}\hat{a}^{\dagger}_{2\text{out}} + e^{i\phi_4}\hat{a}^{\dagger}_{3\text{out}} + ie^{i\phi_4}\hat{a}^{\dagger}_{4\text{out}}]\\ &[e^{i\phi_3}\hat{a}^{\dagger}_{1\text{out}} + ie^{i\phi_3}\hat{a}^{\dagger}_{2\text{out}} + ie^{i\phi_4}\hat{a}^{\dagger}_{3\text{out}} - e^{i\phi_4}\hat{a}^{\dagger}_{4\text{out}}]\,|0000\rangle\end{aligned} \tag{S11}$$

where $\hat{a}^{\dagger}_{i\text{out}}$ represents the creation operator for the i-th output port.

$$\begin{aligned}|\psi\rangle_{out} =& \frac{1}{16}[e^{i2\phi_1}(\hat{a}^{\dagger}_{1\text{out}} - i\hat{a}^{\dagger}_{2\text{out}})(\hat{a}^{\dagger}_{1\text{out}} - i\hat{a}^{\dagger}_{2\text{out}}) + e^{i2\phi_2}(\hat{a}^{\dagger}_{3\text{out}} - i\hat{a}^{\dagger}_{4\text{out}})(\hat{a}^{\dagger}_{3\text{out}} - i\hat{a}^{\dagger}_{4\text{out}})]\\ &[e^{i2\phi_3}(\hat{a}^{\dagger}_{1\text{out}} + i\hat{a}^{\dagger}_{2\text{out}})(\hat{a}^{\dagger}_{1\text{out}} + i\hat{a}^{\dagger}_{2\text{out}}) + e^{i2\phi_4}(\hat{a}^{\dagger}_{3\text{out}} + i\hat{a}^{\dagger}_{4\text{out}})(\hat{a}^{\dagger}_{3\text{out}} + i\hat{a}^{\dagger}_{4\text{out}})]\,|0000\rangle\\ &= \frac{1}{16}e^{2i(\phi_1+\phi_3)}[(\hat{a}^{\dagger}_{1\text{out}})^4 + (\hat{a}^{\dagger}_{2\text{out}})^4 + 2(\hat{a}^{\dagger}_{1\text{out}})^2(\hat{a}^{\dagger}_{2\text{out}})^2]\,|0000\rangle\\ &+ \frac{1}{16}[e^{2i(\phi_1+\phi_4)} + e^{2i(\phi_2+\phi_3)}][(\hat{a}^{\dagger}_{1\text{out}})^2(\hat{a}^{\dagger}_{3\text{out}})^2 - (\hat{a}^{\dagger}_{1\text{out}})^2(\hat{a}^{\dagger}_{4\text{out}})^2\\ &- (\hat{a}^{\dagger}_{2\text{out}})^2(\hat{a}^{\dagger}_{3\text{out}})^2 + (\hat{a}^{\dagger}_{2\text{out}})^2(\hat{a}^{\dagger}_{4\text{out}})^2 + 4\hat{a}^{\dagger}_{1\text{out}}\hat{a}^{\dagger}_{2\text{out}}\hat{a}^{\dagger}_{3\text{out}}\hat{a}^{\dagger}_{4\text{out}}]\,|0000\rangle\\ &+ \frac{1}{8}i[e^{2i(\phi_1+\phi_4)} - e^{2i(\phi_2+\phi_3)}][(\hat{a}^{\dagger}_{1\text{out}})^2\hat{a}^{\dagger}_{3\text{out}}\hat{a}^{\dagger}_{4\text{out}} - (\hat{a}^{\dagger}_{2\text{out}})^2\hat{a}^{\dagger}_{3\text{out}}\hat{a}^{\dagger}_{4\text{out}}\\ &- \hat{a}^{\dagger}_{1\text{out}}\hat{a}^{\dagger}_{2\text{out}}(\hat{a}^{\dagger}_{3\text{out}})^2 + \hat{a}^{\dagger}_{1\text{out}}\hat{a}^{\dagger}_{2\text{out}}(\hat{a}^{\dagger}_{4\text{out}})^2]\,|0000\rangle\\ &+ \frac{1}{16}e^{2i(\phi_2+\phi_4)}[(\hat{a}^{\dagger}_{3\text{out}})^4 + (\hat{a}^{\dagger}_{4\text{out}})^4 + 2(\hat{a}^{\dagger}_{3\text{out}})^2(\hat{a}^{\dagger}_{4\text{out}})^2]\,|0000\rangle\\ &= \frac{1}{8}e^{2i(\phi_1+\phi_3)}[\sqrt{6}(|4000\rangle + |0400\rangle) + 2\,|2200\rangle]\\ &+ \frac{1}{8}e^{2i(\phi_2+\phi_4)}[\sqrt{6}(|0040\rangle + |0004\rangle) + 2\,|0022\rangle]\\ &+ \frac{1}{8}[e^{2i(\phi_1+\phi_4)} + e^{2i(\phi_2+\phi_3)}][|2020\rangle - |2002\rangle - |0220\rangle + |0202\rangle + 2\,|1111\rangle]\\ &+ \frac{\sqrt{2}i}{8}[e^{2i(\phi_1+\phi_4)} - e^{2i(\phi_2+\phi_3)}][|2011\rangle - |0211\rangle - |1120\rangle + |1102\rangle]\end{aligned} \tag{S12}$$

where $|i, j, k, l\rangle$ is the symmetric superposition of the four-photon states corresponding to (i, j, k, l) photons exiting in the four output ports. Therefore, when $\phi_1 = \phi_2 = \phi_3 = \phi_4 = 0$, the output quantum state can be simplified as

$$\begin{aligned} |\psi\rangle_{out} = &\frac{\sqrt{6}}{8}(|4000\rangle + |0400\rangle + |0040\rangle + |0004\rangle) \\ &+ \frac{1}{4}(|2200\rangle + |0022\rangle + |2020\rangle - |2002\rangle - |0220\rangle + |0202\rangle + 2\,|1111\rangle) \end{aligned} \tag{S13}$$

when $\phi_1 = \pi/2, \phi_2 = \phi_3 = \phi_4 = 0$, the output quantum state can be simplified as

$$\begin{aligned} |\psi\rangle_{out} = &\frac{\sqrt{6}}{8}(|4000\rangle + |0400\rangle - |0040\rangle - |0004\rangle + 2\,|2200\rangle - 2\,|0022\rangle) \\ &+ \frac{\sqrt{2}i}{4}(|2011\rangle - |0211\rangle - |1120\rangle + |1102\rangle) \end{aligned} \tag{S14}$$
